# Mammalian pexophagy at a glance

**DOI:** 10.1242/jcs.259775

**Published:** 2024-05-16

**Authors:** Justyna Bajdzienko, Anja Bremm

**Affiliations:** Goethe University Frankfurt, Medical Faculty, Institute of Biochemistry II, Theodor-Stern-Kai 7, 60590 Frankfurt am Main, Germany

**Keywords:** Peroxisome, Pexophagy, Selective autophagy, Ubiquitylation

## Abstract

Peroxisomes are highly plastic organelles that are involved in several metabolic processes, including fatty acid oxidation, ether lipid synthesis and redox homeostasis. Their abundance and activity are dynamically regulated in response to nutrient availability and cellular stress. Damaged or superfluous peroxisomes are removed mainly by pexophagy, the selective autophagy of peroxisomes induced by ubiquitylation of peroxisomal membrane proteins or ubiquitin-independent processes. Dysregulated pexophagy impairs peroxisome homeostasis and has been linked to the development of various human diseases. Despite many recent insights into mammalian pexophagy, our understanding of this process is still limited compared to our understanding of pexophagy in yeast. In this Cell Science at a Glance article and the accompanying poster, we summarize current knowledge on the control of mammalian pexophagy and highlight which aspects require further attention. We also discuss the role of ubiquitylation in pexophagy and describe the ubiquitin machinery involved in regulating signals for the recruitment of phagophores to peroxisomes.

## Teaser

Summary: We review current knowledge of how pexophagy, the selective degradation of peroxisomes, is induced and regulated in mammalian cells, and discuss remaining gaps in the understanding of this pathway.

## Introduction

Peroxisomes play essential roles in cellular metabolism (Wanders et al., 2023), and they are important mediators of adaptive responses to metabolic and environmental stress (He et al., 2021). Mutations in genes encoding peroxisomal proteins and decline of peroxisome activity with age are associated with human disease and genetic disorders (see Box 1). Recent studies have also substantiated the role of peroxisomes in host immune signalling and viral infections (Cook et al., 2019; Ferreira et al., 2022). To fulfil these functions, peroxisomes require continued interactions with other organelles, such as the endoplasmic reticulum (ER), mitochondria, lipid droplets and lysosomes (Schrader et al., 2020; Wanders et al., 2016).

**Box 1. Peroxisomal disorders**

Peroxisomal function is indispensable for life. Human peroxisomes contain more than 50 distinct enzymatic activities, most of which are catalysed by unique peroxisomal proteins. Variants in genes encoding peroxisomal proteins can result in various peroxisomal disorders, either affecting specific metabolic pathways (single peroxisomal enzyme deficiencies, PEDs) or causing a generalized defect in the function and assembly of peroxisomes (peroxisome biogenesis disorders, PBDs) (Waterham et al., 2016). The detrimental consequences of defective peroxisomes on human health originate from aberrant development of the brain, demyelination, loss of axonal integrity, neuroinflammation or other neurodegenerative processes (Wanders et al., 2023).

PBDs are a genetically heterogeneous group of autosomal recessive inherited disorders. They are mainly caused by biallelic mutations in any of the PEX genes encoding peroxins, which are proteins required for the import of peroxisomal membrane or matrix proteins and for peroxisome division. The most prominent PBDs are Zellweger spectrum disorders, including Zellweger syndrome (ZS), neonatal adrenoleukodystrophy and infantile Refsum disease. Individuals born with ZS typically die within the first year of life (Waterham et al., 2016). PEX13-deficient mice exhibit many clinical features of ZS and die during the neonatal period (Maxwell et al., 2003). Interestingly, loss of receptor export module (REM) activity due to common variants in individuals suffering from PBDs, such as PEX1 (G843D), triggers pexophagy and, thereby, excessive peroxisome removal (Law et al., 2017). For more detailed accounts of PBDs and their molecular pathogenesis see recent reviews (Fujiki et al., 2020; Wanders et al., 2023; Waterham et al., 2016).

Peroxisomal functions also decline during ageing, a process that likely contributes to the early onset of multiple age-related neurodegenerative disorders, such as Alzheimer's disease and amyotrophic lateral sclerosis, as well as to the pathogenesis of diabetes and cancer (Cipolla and Lodhi, 2017). Therefore, the possibility of targeting peroxisomal function or pexophagy for disease prevention or treatment is intriguing but still requires further exploration. A recent study supports this vision by showing that drugs designed to disrupt peroxisome homeostasis may serve as unconventional therapies to combat resistance in cancer (Dahabieh et al., 2022).

**Figure:**
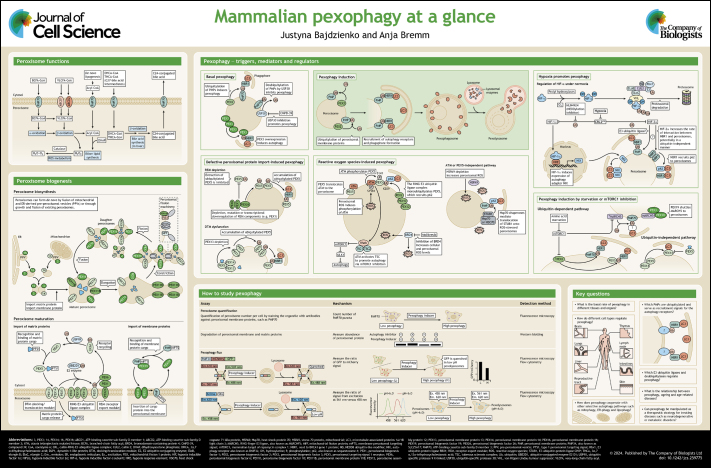
See Supplementary information for a high-resolution version of the poster.

Mammalian cells contain several hundred to thousands of peroxisomes, which vary in shape, size and distribution (Grabenbauer et al., 2000; Hoffman et al., 2020). Peroxisomes display significant heterogeneity in metabolic functions, protein content and number amongst different organisms and cell types. Regardless of this diversity, peroxisomes share a core set of conserved peroxin (PEX) proteins critical for peroxisome biogenesis. Peroxisomes can form *de novo* by fusion of mitochondrial and ER-derived pre-peroxisomal vesicles (PPVs) (Mayerhofer et al., 2016; Sugiura et al., 2017) or through growth and division of existing peroxisomes (see poster) (Schrader et al., 2016). Maintenance of peroxisome homeostasis is a dynamic process that balances biogenesis and degradation; for instance, cultured CHO cells have a peroxisome half-life of ∼2 days under basal growth conditions (Huybrechts et al., 2009). Peroxisome turnover dynamics might vary in different human cell types and require further investigation. Peroxisomes are primarily removed by a selective autophagy process (see Box 2) called pexophagy, which targets peroxisomes to lysosomes for degradation (Iwata et al., 2006). In this Cell Science at a Glance article and the accompanying poster, we describe the current understanding of how pexophagy is induced and regulated in mammalian cells, and which E3 ubiquitin ligases and deubiquitylases (DUBs) are involved in this process. We will address open questions in the mammalian pexophagy research field and present an overview of tools and technological advances that can be used to tackle these questions.

**Box 2. Selective autophagy**

Selective autophagy targets specific cellular components, such as proteins or organelles, as well as pathogens, for lysosomal degradation. This process is triggered under conditions of nutrient or growth factor deficiency, or in response to various cellular stresses. Autophagy plays a crucial role in maintaining cellular homeostasis, and dysregulation of the autophagic pathway has been implicated in the pathophysiology of various human diseases, including cancer, neurodegeneration and infections (Klionsky et al., 2021). Selective autophagy mainly uses ubiquitin as a signal to recruit autophagy receptors containing ubiquitin-binding domains, such as p62 (also known as sequestosome-1, SQSTM1), next to BRCA1 gene 1 protein (NBR1) or optineurin (OPTN), to specific cargo destined for autophagic degradation (Johansen and Lamark, 2020). These receptors, in turn, attach to lipidated microtubule-associated proteins 1A/1B light chain 3 (LC3) molecules, which are anchored in the phagophore membrane, through their LC3-interacting region (LIR), thereby facilitating engulfment of the cargo by the phagophore. Once the autophagosome is formed, it fuses with a lysosome, creating an autolysosome, where the degradation process occurs (Chang et al., 2021). For detailed information about the molecular mechanisms of autophagosome formation and the core autophagy machinery, including the role of ubiquitin-like LC3 and GABARAP-family members, we refer readers to recent in-depth reviews (Chang et al., 2021; Dikic and Elazar, 2018; Nakatogawa, 2020).

## Peroxisome dynamics and pexophagy

Peroxisomes are dynamic, highly plastic organelles capable of adjusting their morphology, number, intracellular position, inter-organellar interactions and metabolic functions according to the needs of the cell or organism (Wanders et al., 2023). New peroxisomes form by growth and division or multiplication, and superfluous organelles are degraded by pexophagy. Because impaired peroxisomal protein import, and therefore organelle maturation, is a major inducer of pexophagy, we will describe peroxisome biogenesis here in more detail. Peroxisomes divide through elongation, constriction and fission mediated by PEX11B, dynamin-1-like protein (DLP1), and the two mitochondrial fission factors MFF and FIS1 (see poster) (Carmichael et al., 2022). In addition, new peroxisomes form *de novo* by selective release of PPVs containing PEX3 and PEX14 from mitochondria, which fuse with ER-derived vesicles carrying PEX16, producing organelles competent for peroxisomal membrane protein (PMP) and luminal enzyme import (see poster) (Sugiura et al., 2017). Other studies have shown that PEX3 is also targeted co-translationally into the ER membrane, where it buds off in vesicles that mature into peroxisomes (Mayerhofer et al., 2016; Schrul and Kopito, 2016). A more detailed description of *de novo* biogenesis of peroxisomes has recently been published (Banerjee and Prinz, 2023).

Peroxisomal proteins are synthesized by cytosolic free ribosomes and contain a peroxisomal targeting signal (PTS) that determines their cellular destination. Most PMPs are recognized by the receptor PEX19 via an internal binding motif (referred to as an mPTS). PMP-loaded PEX19 shuttles to peroxisomes and docks to the transmembrane protein PEX3, which in turn is bound to PEX16, followed by insertion of the PMP into the membrane (see poster) (Fang et al., 2004; Jones et al., 2004; Matsuzaki and Fujiki, 2008). Functional loss of PEX3, PEX16 or PEX19 results in cells completely lacking peroxisomal membranes (Kim, 2017).

Most peroxisomal matrix proteins contain a type 1 PTS (PTS1; typically ending in the amino acid sequence SKL) at the C terminus, which is recognized by the shuttling receptor PEX5 (Stanley et al., 2006). A few additional peroxisomal matrix proteins are targeted to peroxisomes by a type 2 PTS at their N termini, recognized by the import receptor PEX7, which requires PEX5 as a co-receptor (Schliebs and Kunau, 2006). Once PEX5 binds to a cargo protein, it is inserted into a transmembrane complex known as the docking/translocation module (DTM), which includes PEX13 and PEX14. The DTM interacts with the RING E3 ubiquitin ligase complex comprising PEX2, PEX10 and PEX12 (see poster) (Meinecke et al., 2010). Notably, PEX5 transports folded and even oligomeric matrix proteins across the peroxisomal membrane (Gao et al., 2022; Ravindran et al., 2023; Walton et al., 1995). A recent study has found that PEX5 fully accompanies its cargo into the peroxisomal lumen (Skowyra and Rapoport, 2022). The RING E3 ubiquitin ligase complex initiates recycling of PEX5 via monoubiquitylation at a conserved cysteine residue (C11 in mammals) (Carvalho et al., 2007; Platta et al., 2009; Schwartzkopff et al., 2015). This complex is proposed to form a retro-translocation channel for PEX5 (Feng et al., 2022). PEX5 is then extracted back into the cytosol by the AAA+ ATPase proteins PEX1 and PEX6, and the membrane anchor protein PEX26, which together have been termed the receptor export module (REM) (see poster) (Pedrosa et al., 2018). After PEX5 is deubiquitylated, it is ready for a new round of import. The mechanism for deubiquitylation of PEX5 at C11 is not fully clarified, but it is likely a combination of enzymatic and non-enzymatic activities (Apanasets et al., 2014). The DUB USP9X has been linked to this process (Grou et al., 2012). Interestingly, in contrast to other translocases requiring nucleoside triphosphate hydrolysis or a membrane potential, the peroxisomal matrix protein import machinery is driven by strong and multivalent protein–protein interactions between PEX5 and the DTM. ATP hydrolysis is used only for threading PEX5 back into the cytosol by PEX1 and PEX6 (Pedrosa et al., 2019).

K464 modification by the E3 ubiquitin ligase TRIM37 stabilizes PEX5 and promotes peroxisomal matrix protein import (Wang et al., 2017). Loss of TRIM37 results in impaired PTS-mediated protein import and proteasomal degradation of PEX5. However, the corresponding (poly-)ubiquitin signal that targets PEX5 to the proteasome is unknown.

Efficient PEX5 recycling is important for peroxisome homeostasis, and the accumulation of ubiquitylated PEX5 within the peroxisomal membrane due to impaired removal triggers degradation of the organelle by pexophagy (Law et al., 2017; Nordgren et al., 2015). These studies underline that defects in the matrix import machinery are a major trigger for pexophagy, which we discuss in more detail below.

## Selective turnover of peroxisomes by pexophagy

In mammalian cells, several different stimuli that provoke pexophagy have been described, including amino acid starvation and mammalian target of rapamycin complex 1 (mTORC1) inhibition, dysfunctional peroxisomal import machinery, hypoxia, and redox stress. Most of these stimuli provoke ubiquitylation of PMPs, which in turn recruit autophagy receptors that create a bridge from the modified peroxisome cargo to the growing phagophore for degradation in autolysosomes (see poster). Specific ubiquitylated PMPs have been described, and the E3 ubiquitin ligase and/or specific autophagy receptors have been identified for certain pexophagy-inducing stimuli. However, our knowledge is still limited regarding how peroxisome turnover is executed in most conditions.

The pivotal role of PMP ubiquitylation in pexophagy has been established by artificially fusing ubiquitin to PMP34 (also known as SLC25A17) (Kim et al., 2008). A single ubiquitin attached to PMP34 facing the cytosolic side of the peroxisome membrane is sufficient to induce pexophagy in otherwise unperturbed cells. These monoubiquitin signals are recognized by the autophagy receptor NBR1. Additional binding of NBR1 to a second autophagy receptor, p62, significantly increases pexophagy efficiency (Deosaran et al., 2012; Kim et al., 2008). A systematic characterization of ubiquitylation sites on peroxisomes has not yet been performed. The role of polyubiquitylation of PMPs in peroxisome turnover also remains elusive. The following sections will summarize what is known regarding factors required for pexophagy and the mechanisms involved in induction of pexophagy under specific cellular stimuli.

### Basal pexophagy

Under basal conditions, pexophagy can be stimulated by overexpression and targeting to peroxisomes of the PMP import receptor PEX3. Elevated PEX3 levels promote peroxisome ubiquitylation, organelle clustering and degradation in lysosomes (see poster) (Yamashita et al., 2014). PEX3-induced pexophagy is dependent on NBR1 recruitment because knockdown of this autophagy receptor rescues autophagic degradation of peroxisomes. Although the specific ubiquitin signals that are relevant for recruiting NBR1 to PMPs are unknown, PEX3 ubiquitylation is not required, as both wild-type and mutant, lysine-deficient PEX3 equally stimulate pexophagy (Yamashita et al., 2014).

The first DUB to be linked to pexophagy regulation is USP30, a well-characterized regulator of mitophagy (see poster) (Bingol et al., 2014; Cunningham et al., 2015). Ectopic expression of USP30 on the surface of peroxisomes reduces pexophagy in HEK293 cells under basal growth conditions (Cunningham et al., 2015). More detailed studies on USP30 using the pH-sensitive fluorophore Keima targeted to peroxisomes (Keima–SKL) have established that both knockdown and knockout of USP30 increase basal pexophagy in hTERT-RPE1 cells. The enzymatic activity of USP30 is required to counteract pexophagy by deubiquitylating ATP-binding cassette sub-family D member 3 (PMP70), which transports long-branched-chain fatty acids and bile acids from the cytosol into the peroxisomal lumen, as wild-type USP30 but not catalytically inactive USP30 can restore pexophagy to baseline levels in USP30 knockout cells (Marcassa et al., 2018). Pharmacological inhibition of USP30 using compound 39 (CMPD-39) in U2OS cells expressing the pexophagy reporter Keima–SKL also enhances basal pexophagy (Rusilowicz-Jones et al., 2022).

### Pexophagy induction as a consequence of defective peroxisomal protein import

Peroxisome homeostasis is dependent on a flawless peroxisomal import machinery, and disrupted import can stimulate pexophagy. PEX13 is part of the DTM required for import of peroxisomal matrix proteins, and it prevents degradation of healthy peroxisomes. Knockout of PEX13 results in accumulation of ubiquitylated PEX5 and peroxisome-dependent reactive oxygen species (ROS), which promote pexophagy (see poster) (Demers et al., 2023).

After release of PEX5-associated cargo proteins into the lumen of peroxisomes, the REM is crucial for recycling PEX5 back into the cytoplasm. By securing PEX5 availability for further protein import, the REM components PEX1, PEX6 and PEX26 play an active role in preventing excess pexophagy and the development of peroxisome biogenesis disorders (PBDs) (see Box 1 and poster). PEX1 (G843D) is a common variant in individuals suffering from PBDs and triggers excessive pexophagy due to accumulation of ubiquitylated PEX5 in peroxisomes and the subsequent recruitment of NBR1 (Law et al., 2017; Nordgren et al., 2015). Modulation of PEX1 at the transcriptional level also affects peroxisome turnover. The RNA-binding protein HNRNPA1 interacts with the 3′ untranslated region of *PEX1* mRNA and regulates PEX1 expression in HeLa cells, and HNRNPA1 depletion increases the amount of peroxisomal ROS and promotes pexophagy (Park et al., 2021). Interestingly, overexpression of USP30 in HeLa cells depleted of PEX1 or PEX26 rescues peroxisome loss. The same phenotype has been observed in patient-derived PEX1 (G843D) fibroblasts (Riccio et al., 2019); however, it should be noted that peroxisomes in PEX1 (G843D) cells are likely not mature or functional because impaired PEX1 still interferes with normal cargo import (Marcassa et al., 2019). Mechanistically, it is also not clear whether USP30 deubiquitylates PEX5 itself or PMPs downstream of PEX5 accumulation. Nevertheless, the therapeutic potential of USP30 for individuals with PBDs should be investigated.

### Pexophagy induction by starvation or mTORC1 inhibition

Another potent inducer of pexophagy is amino acid starvation and, accordingly, inhibition of mTORC1. Although peroxisomes can be degraded non-specifically by macroautophagy under these conditions, amino acid starvation also causes cells to actively downregulate PEX13 and triggers ubiquitylation of PEX5 and PMP70 by PEX2, which in turn serves as a signal for recruitment of NBR1 to peroxisomes and induces selective autophagy via pexophagy (see poster) (Demers et al., 2023; Sargent et al., 2016). Interestingly, amino acid starvation or rapamycin treatment, which inhibits mTORC1, upregulates PEX2 expression, suggesting a positive feedback mechanism that further increases pexophagy.

The E3 ubiquitin ligase MARCH5 (also known as MARCHF5), which is present in mitochondria and peroxisomes, has also been associated with starvation-induced pexophagy (Zheng et al., 2022). It is targeted by the PMP shuttling receptor PEX19 to peroxisomes, where it ubiquitylates PMP70. Depletion of MARCH5 impairs PMP70 ubiquitylation and pexophagy induction upon mTORC1 inhibition (see poster) (Zheng et al., 2022). Further research is required to fully understand the function of PEX2 and MARCH5 in the context of pexophagy mediated by amino acid starvation and mTORC1 inhibition, as it is unknown whether they play complementary or redundant roles. As mentioned above, a comprehensive picture of which PMPs are ubiquitylated by these E3 ligases has not been described. Are just a few specific PMPs ubiquitylated, or do E3 ligases decorate peroxisomes with a ‘ubiquitin coat’ reminiscent of how parkin ubiquitylates damaged mitochondria (Harper et al., 2018)? Overexpression of USP30 also counteracts amino acid starvation-induced pexophagy (Riccio et al., 2019). In contrast, whereas USP30 depletion promotes basal peroxisome turnover, it does not affect starvation-induced pexophagy. The signals for starvation-induced degradation are potentially sufficiently strong that turnover is not further elevated by removing USP30. Interestingly, co-depletion of USP30 and PEX2 prevents both starvation-induced and basal pexophagy (Riccio et al., 2019), suggesting that USP30 deubiquitylates PEX2 substrates in both scenarios.

Serum withdrawal also induces pexophagy. Under serum-starvation conditions in CHO-K1 cells, PEX14, which is part of the DTM, directly binds to the lipidated form of LC3 (LC3-II), thereby promoting autophagosome engulfment of the peroxisome without the requirement for PMP ubiquitylation (see poster) (Jiang et al., 2015). PEX14 does not contain an LIR but interacts with LC3-II via its transmembrane domain, an interaction that is competitively inhibited by PEX5.

### Pexophagy induced by oxidative stress

Increases in cytosolic and peroxisomal ROS levels also provoke pexophagy. Catalase is a peroxisomal matrix protein and H_2_O_2_-decomposing enzyme, and its knockdown or inhibition by 3-aminotriazole (3-AT) promotes pexophagy upon serum starvation. Specifically, 3-AT treatment induces NBR1-dependent autophagy, PEX5 ubiquitylation and ROS accumulation in peroxisomes (Lee et al., 2018). Of note, in response to H_2_O_2_ accumulation in the cytosol, PEX14 is phosphorylated, which suppresses peroxisomal import of catalase. Consequently, cytosolic catalase levels rise to counteract H_2_O_2_, thereby securing cell survival (Okumoto et al., 2020). This can result in elevated ROS levels within peroxisomes from β-oxidation of fatty acids, which in turn promotes pexophagy induction. The PEX14 phosphorylation-dependent control of catalase import underscores the central role of the DTM peroxins in peroxisome homeostasis and regulation of pexophagy.

The chaperone mortalin (HSPA9, also known as stress-70 protein, mitochondrial) also restricts peroxisomal ROS levels. Knockdown of HSPA9 in both HeLa cells and neuroblastoma SH-SY5Y cells induces ROS- and p62-dependent pexophagy (Jo et al., 2020). Specific ubiquitin signals have not been described yet in this context. Interestingly, in contrast to wild-type HSPA9, a Parkinson’s disease-derived variant of HSPA9 fails to rescue pexophagy in HSPA9-depleted cells (Jo et al., 2020), raising the question of whether loss-of-function variants of HSPA9 affect pexophagy and if, given the functional interaction between both organelles, degradation of peroxisomes also impacts turnover of mitochondria in individuals with Parkinson's disease.

The tumour suppressor TSC is a heterodimer comprising hamartin (TSC1) and the GTPase-activating protein (GAP) tuberin (TSC2). TSC inhibits the activity of the small GTPase Rheb at lysosomes to suppress mTORC1 signalling (Settembre and Perera, 2024). TSC1 and TSC2 are also shuttled to peroxisomes by PEX19 and PEX5, respectively (Zhang et al., 2013). PEX5 also localizes ataxia telangiectasia mutated (ATM) kinase to the peroxisome. Peroxisomal ROS activates ATM, which then phosphorylates PEX5 at S141; this triggers subsequent monoubiquitylation of PEX5 at K209. This ubiquitin signal induces pexophagy by recruiting the autophagy receptor p62 (Zhang et al., 2015). In response to peroxisomal ROS, ATM further activates TSC2, resulting in mTORC1 inhibition and increased autophagic flux. Interestingly, inhibition of bromodomain-containing protein 4 (BRD4) by molibresib, a bromodomain and extra-terminal domain (BET) protein small-molecule inhibitor, increases peroxisomal ROS, promotes ATM-dependent phosphorylation of PEX5 and induces pexophagy (see poster) (Kim et al., 2022).

A recent study has shown that E3 ubiquitin-protein ligase CHIP (STUB1) localizes to artificially ROS-stressed peroxisomes to mediate ubiquitin-dependent pexophagy (Chen et al., 2020). STUB1, ubiquitin, p62 and LC3B (MAP1LC3B) accumulate on peroxisomes as a result of ROS induction. In this pathway, members of the 70 kDa heat shock protein family (Hsp70 proteins and HSC70, also known as HSPA8) recognize ROS-stressed peroxisomes and mediate the translocation of STUB1 onto peroxisomes, which promotes pexophagy independently of ATM activity and PEX5 phosphorylation and ubiquitylation. The physiological relevance and the mechanistic details of this pathway need to be investigated further. Moreover, whether the method of peroxisomal ROS induction, as well as the chosen cell model, affects peroxisome turnover by pexophagy must be clarified. For example, it has recently been suggested that increased peroxisomal H_2_O_2_ emission does not promote pexophagy in HEK293 and HeLa cells, but does inhibit autophagy (Li et al., 2023).

### Hypoxia promotes pexophagy

Hypoxia is a condition in which the oxygen consumption of a cell exceeds its oxygen supply. Cellular adaptation to hypoxia is mainly orchestrated by heterodimeric transcription factors known as hypoxia-inducible factors (HIFs) (see poster). The α subunits of HIFs [HIF-α subunits; namely HIF-1α (HIF1A) and HIF-2α (EPAS1)] are carefully regulated to prevent inappropriate target gene expression. In normoxia, HIF-α subunits undergo rapid turnover via an oxygen-dependent enzymatic cascade involving prolyl hydroxylases (PHDs) and a specialized cullin–RING E3 ubiquitin ligase complex (CRL2^VHL^) that includes the von Hippel–Lindau disease tumour suppressor (VHL). PHD-mediated hydroxylation of HIF-α subunits targets them for recognition by VHL and proteasomal degradation. In hypoxia, the PHDs are gradually inhibited, and HIF-α subunit protein levels increase (Jaakkola et al., 2001; Ivan et al., 2001).

HIF signalling has been linked to peroxisome homeostasis and metabolism. Liver peroxisome abundance is reduced in *Vhl^−/−^* mice and can be rescued by autophagy inhibition (Walter et al., 2014). Specifically, HIF-2α activation in hepatocytes promotes peroxisome turnover via pexophagy in an NBR1-dependent manner, although the E3 ubiquitin ligase involved in this pathway has not yet been determined (see poster). In clear cell renal cell carcinoma (ccRCC), increased HIF-2α levels due to loss of VHL function correlate with low peroxisome numbers, further establishing HIF-2α as negative regulator of peroxisome homeostasis (Walter et al., 2014).

HIF-1α has also been associated with regulation of pexophagy (Wilhelm et al., 2022). CRL2^VHL^ must be NEDDylated to become active. The compound MLN4924 is a NEDDylation inhibitor that triggers apoptosis, cell cycle arrest and autophagy. In the presence of MLN4924, CRL2^VHL^ enzymatic activity is inhibited and HIF1-α is activated and binds to hypoxia-response elements (HREs); this induces expression of the adaptor protein NIX (also known as BNIP3L) and, thereby, degradation of peroxisomes via selective autophagy (Barone et al., 2023). Furthermore, under iron-chelating or hypoxic conditions, HIF1-α is stabilized and NIX expression is induced, which coordinates both mitophagy and pexophagy. NIX localizes to peroxisomes and directly interacts with LC3 to induce pexophagy (see poster). In this scenario, PMP ubiquitylation is not required. Interestingly, reduced peroxisome numbers have been observed in the retina of NIX knockout mice via quantification of PMP70 and catalase, supporting a significant additional role for NIX in regulation of pexophagy under normoxic conditions (Wilhelm et al., 2022).

## How to study pexophagy in mammalian cells

Several tools and methods have been developed to study pexophagy in mammalian cells (see poster). To follow pexophagy, the levels of peroxisomal marker proteins can be monitored by western blotting. For analysis of peroxisome dynamics, peroxisomes can be visualized by antibody staining, by targeting fluorescent proteins to the peroxisome matrix, by directly fusing fluorophores to PMPs or by other labelling methods such as the HaloTag system (Huybrechts et al., 2009). However, many peroxisomal proteins exhibit only marginally decreased levels or undergo relocation to the cytoplasm during pexophagy (Okumoto et al., 2020; Walton et al., 2017), making it difficult to use them to establish quantitative measures of pexophagy or the localization of peroxisomal proteins in autophagolysosomes. To overcome these limitations, specific pexophagy reporters have been established, such as Keima–SKL (Barone et al., 2023; Cunningham et al., 2015; Marcassa et al., 2018) or tandem PMP– mCherry–GFP fluorophore constructs targeted to the peroxisomal membrane (Deosaran et al., 2012; Liang et al., 2018), which can both be combined with fluorescence microscopy, live-cell imaging and flow cytometry. Because peroxisome size is at or below the diffraction limit, image-based analysis of the internal organization and dynamics of peroxisomes requires the use of super-resolution microscopy. A recent summary of different super-resolution microscopy approaches highlights the potential for their use in peroxisome research (Galiani et al., 2023).

The genetically encoded photosensitizer KillerRed–SKL generates ROS upon illumination with green light (Bulina et al., 2006), allowing the assessment of pexophagy induction by ROS in individually stressed peroxisomes (Chen and Yang, 2023; Chen et al., 2020). The potential influences of peroxisome transport, positioning within the cell, or peroxisomal protein trafficking and import on pexophagy could also be addressed using optogenetic methods (Spiltoir et al., 2016; van Bergeijk et al., 2015). Furthermore, incorporation of designer non-canonical amino acids (ncAAs) into PMPs, for example to covalently stabilize transient protein–protein interactions at the surface of peroxisomes, can be used to monitor recruitment of the autophagy machinery to specific peroxisomes or the role of specific post-translational modifications (PTMs). Proximity-triggered and residue-selective photo-induced crosslinking approaches, as well as site-specific incorporation of ncAAs mimicking PTMs, are also promising avenues to test how pexophagy is executed and regulated (Krauskopf and Lang, 2020). Proteomic approaches can determine peroxisome content, interaction networks and PTM profiles in the context of pexophagy induction in combination with peroxisome enrichment strategies such as affinity purification using the PEROXO-tag (Ray et al., 2020) or subcellular fractionation (Manner and Islinger, 2023). CRISPR-Cas9 technology has successfully been applied to functionally interrogate the entire genome in the autophagy research field (Liang and Corn, 2022) and has the potential to identify factors that are specifically implicated in pexophagy. However, to examine pexophagy in the context of specific tissues and organs, and to complement data obtained in cultured cells, studies using animal models such as mouse or *Caenorhabditis elegans* are required.

## Concluding remarks and future directions

Although our knowledge of peroxisome physiology, pexophagy and the role of peroxisomes in disease has significantly increased in recent years (Wanders et al., 2023), there are numerous insufficiently answered questions and unknown details regarding mammalian pexophagy that need to be addressed (see poster). For example, the signals or cellular circumstances that specifically induce pexophagy in mammalian cells require further investigation. It remains to be determined which PMPs are ubiquitylated under different stimuli and serve as recruitment signals for the autophagy machinery. Which E3 ubiquitin ligases and DUBs attach and terminate these ubiquitin signals, respectively? Neither the basal rate of pexophagy in different tissues and organs nor whether there are cell type-specific regulation mechanisms of pexophagy is known. In general, it is important to clarify how knowledge about pexophagy generated using standard two-dimensional cell cultures holds true for *in vivo* models. Future studies will also need to elucidate how pexophagy cooperates with other organellophagy pathways such as mitophagy, ER-phagy and lipophagy. Ultimately, the relationship between pexophagy, ageing and age-related diseases needs to be defined, as well as whether the manipulation of pexophagy can be used as a therapeutic strategy for treating diseases such as neurodegenerative disorders or metabolic disorders. Tackling these questions will reveal important new insights into how cells secure peroxisome homeostasis and might uncover possibilities of how pexophagy could be exploited for therapy or disease prevention.

## Poster

Poster

## Panel 1. Peroxisome functions

Panel 1. Peroxisome functions

## Panel 2. Peroxisome biosynthesis

Panel 2. Peroxisome biosynthesis

## Panel 3. Peroxisome maturation

Panel 3. Peroxisome maturation

## Panel 4. Pexophagy induction

Panel 4. Pexophagy induction

## Panel 5. Basal pexophagy

Panel 5. Basal pexophagy

## Panel 6. Defective peroxisomal protein import-induced pexophagy

Panel 6. Defective peroxisomal protein import-induced pexophagy

## Panel 7. Reactive oxygen species-induced pexophagy

Panel 7. Reactive oxygen species-induced pexophagy

## Panel 8. Hypoxia promotes pexophagy

Panel 8. Hypoxia promotes pexophagy

## Panel 9. Pexophagy induction by starvation or mTORC1 inhibition

Panel 9. Pexophagy induction by starvation or mTORC1 inhibition

## Panel 10. How to study pexophagy

Panel 10. How to study pexophagy

## Panel 11. Key questions

Panel 11. Key questions

## High-resolution poster and poster panels

A high-resolution version of the poster and individual poster panels are available for downloading at https://journals.biologists.com/jcs/article-lookup/doi/10.1242/jcs.259775#supplementary-data.
