# Supplementary figures and images for: Mammalian pexophagy at a glance
Source: J Cell Sci. 2024 May 16;137(9):jcs259775. doi: 10.1242/jcs.259775 (PMC11166455; doi:10.1242/jcs.259775)

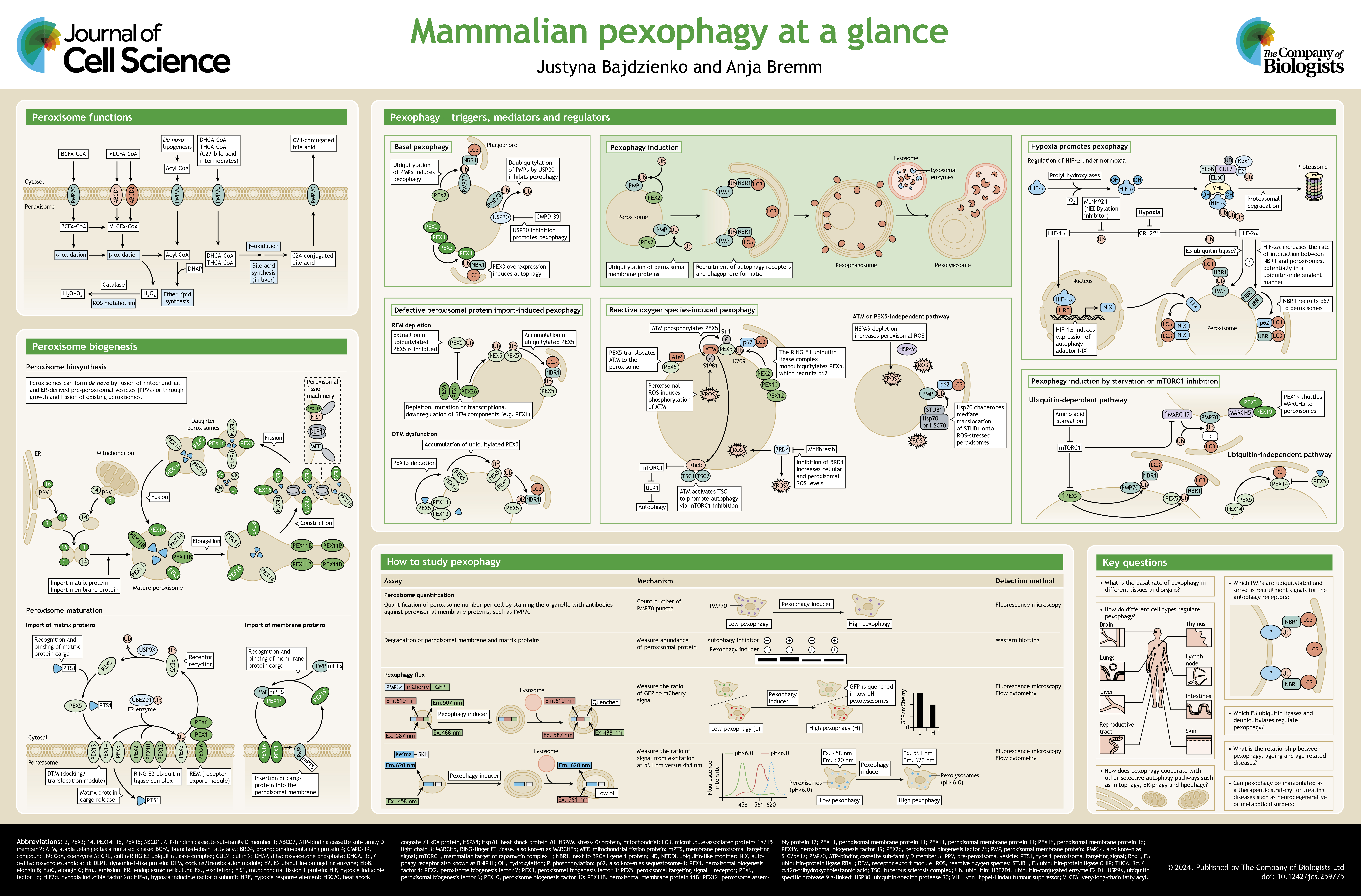

Supplement: Poster [file joces-137-259775-s1.jpg]

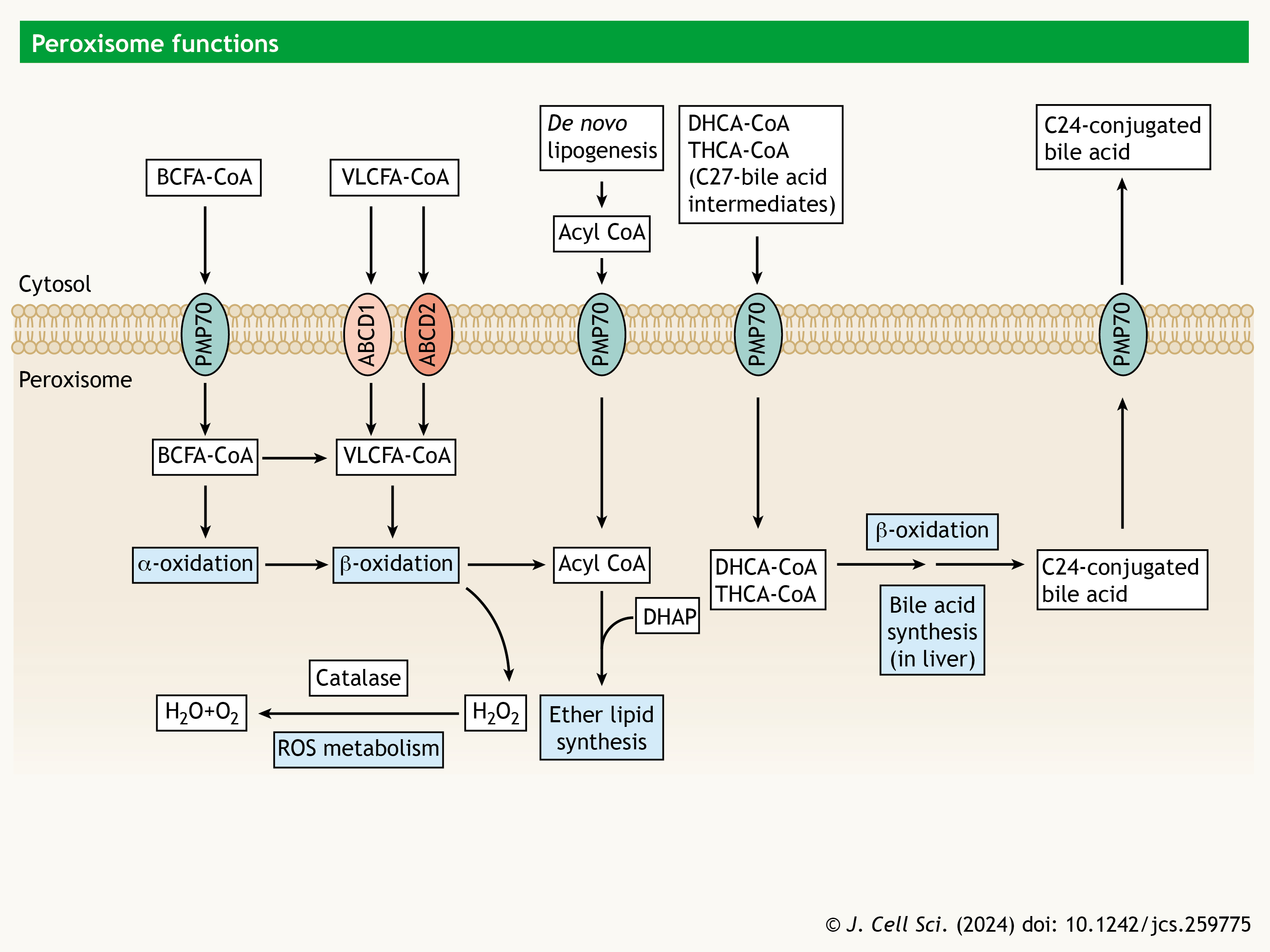

Supplement: Panel 1. Peroxisome functions [file joces-137-259775-s2.jpg]

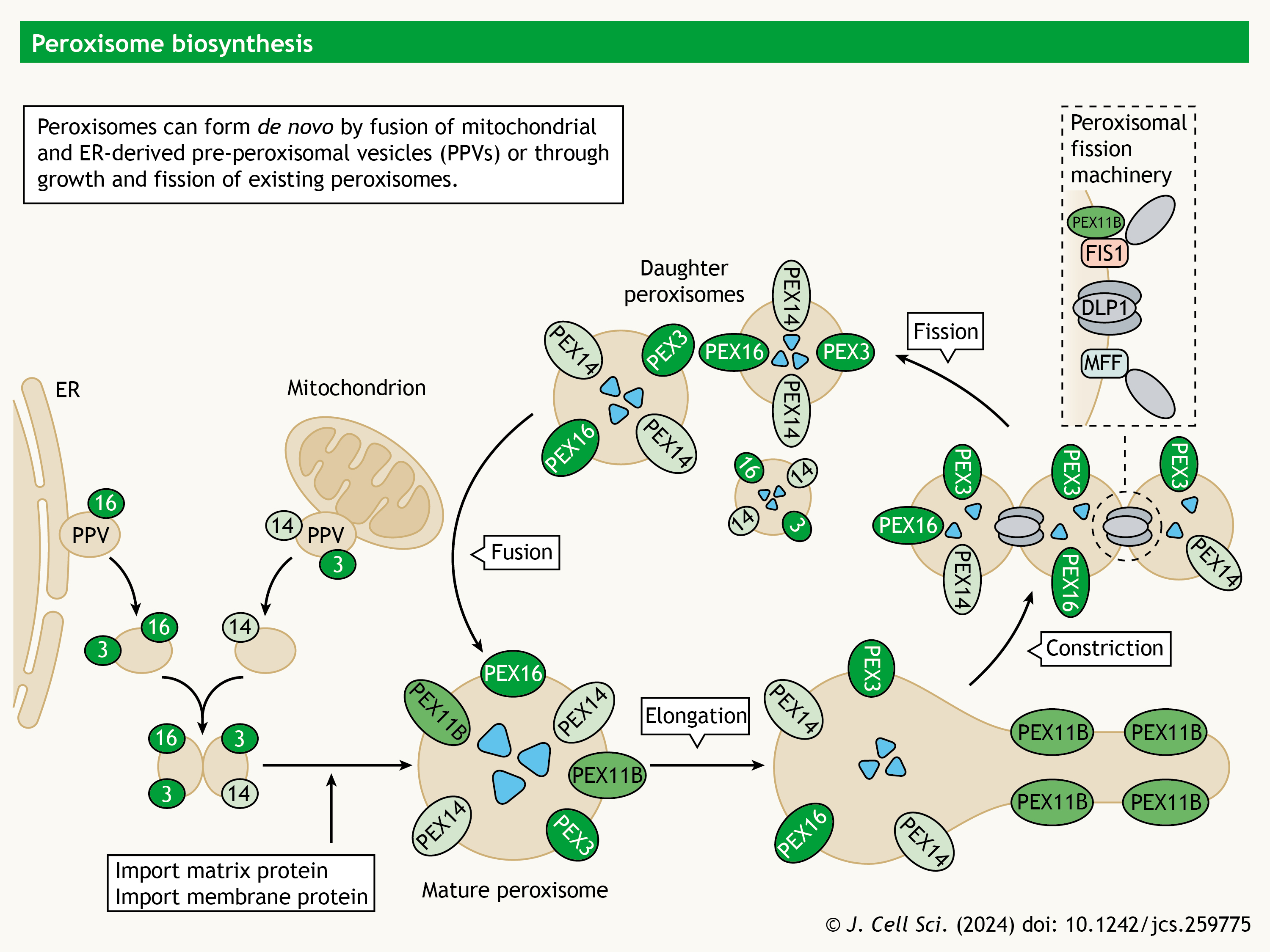

Supplement: Panel 2. Peroxisome biosynthesis [file joces-137-259775-s3.jpg]

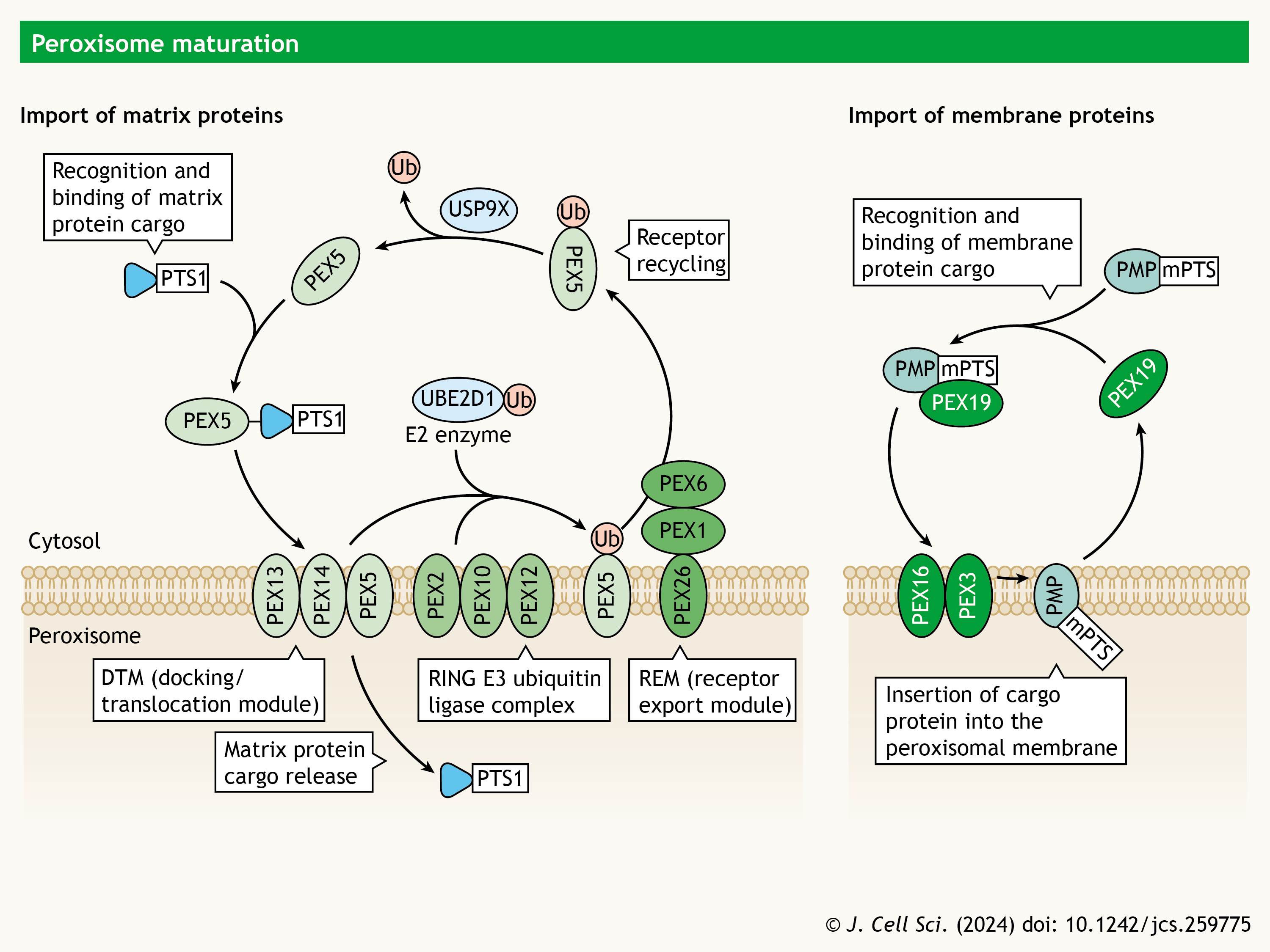

Supplement: Panel 3. Peroxisome maturation [file joces-137-259775-s4.jpg]

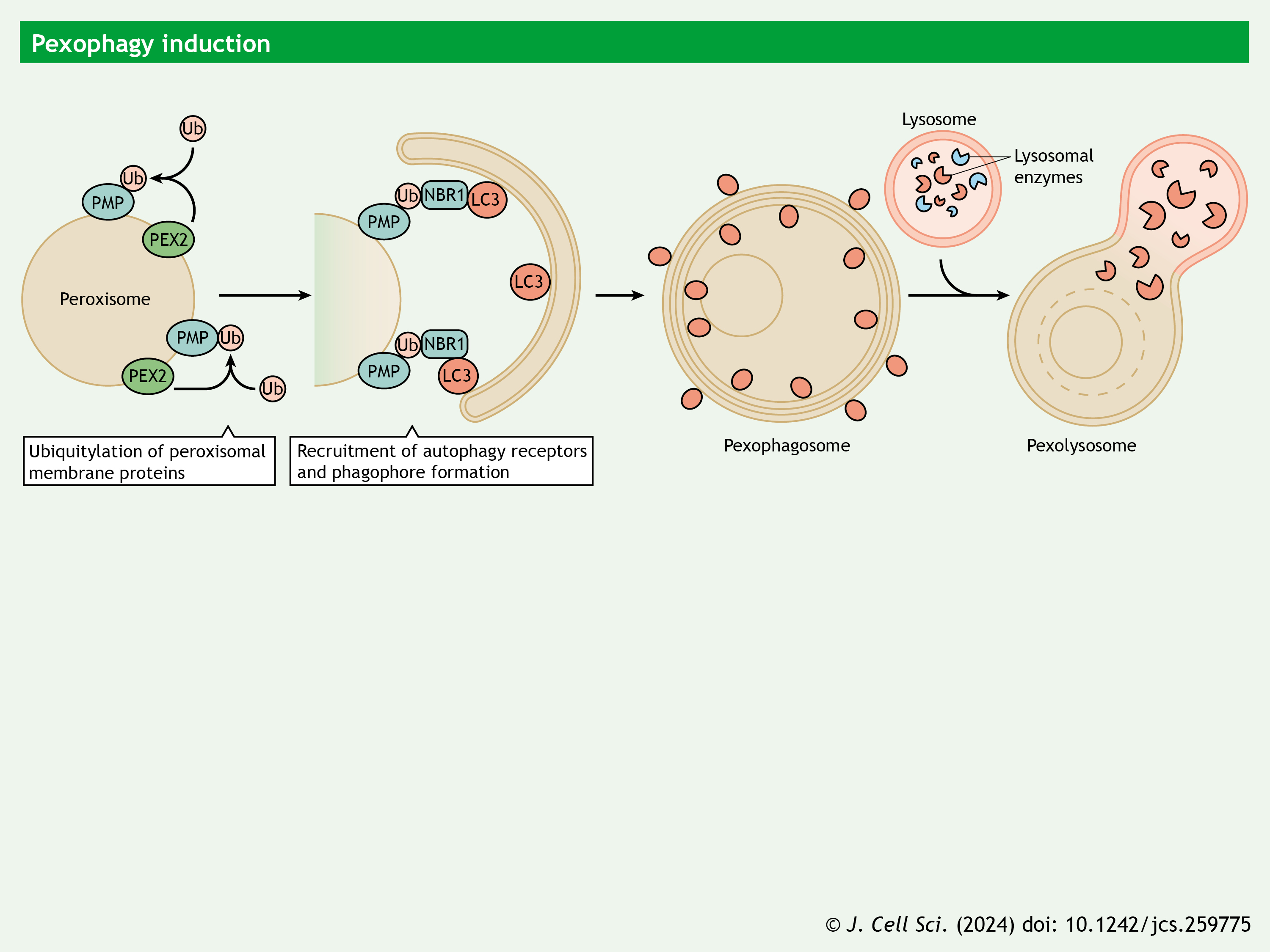

Supplement: Panel 4. Pexophagy induction [file joces-137-259775-s5.jpg]

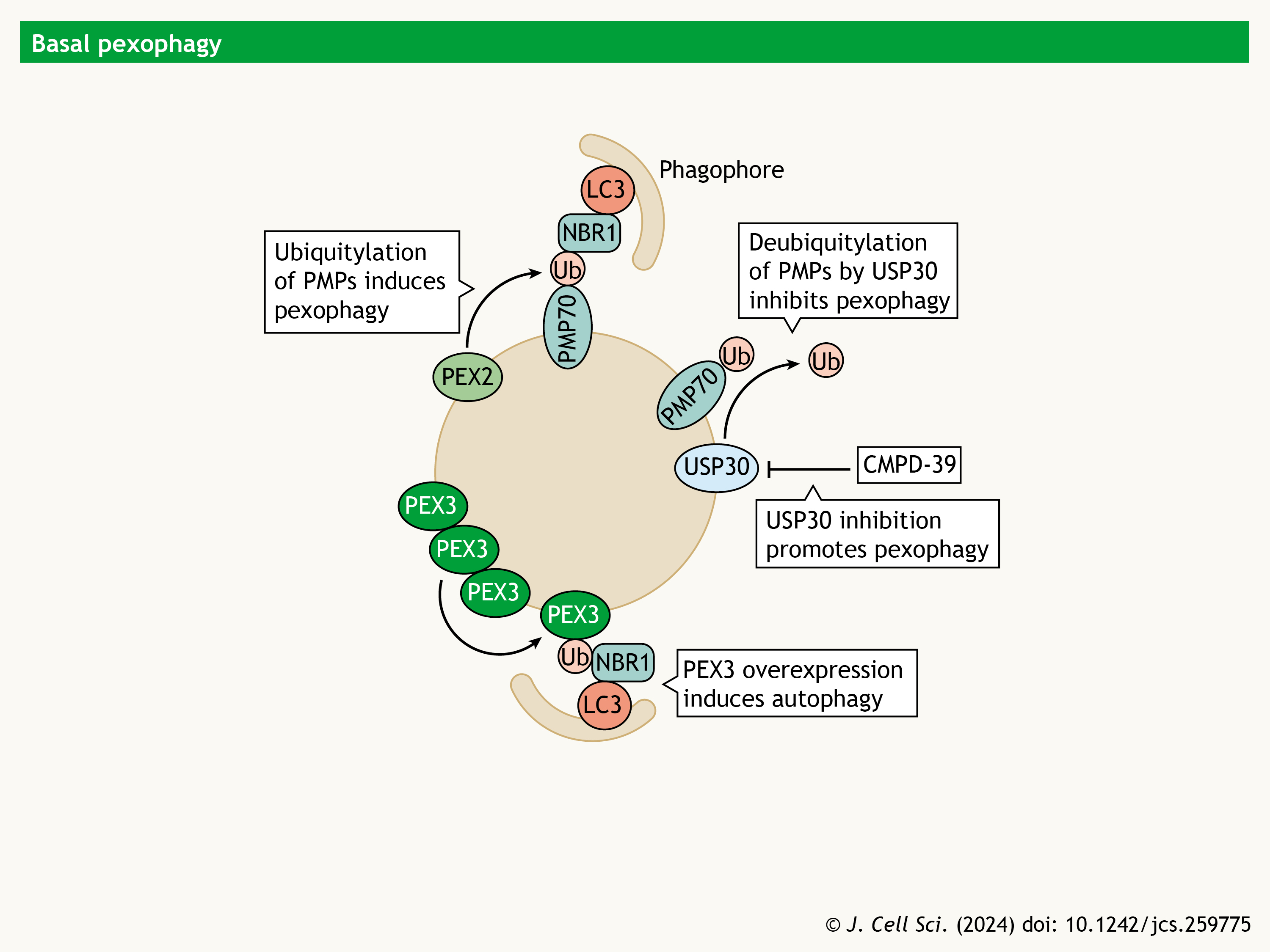

Supplement: Panel 5. Basal pexophagy [file joces-137-259775-s6.jpg]

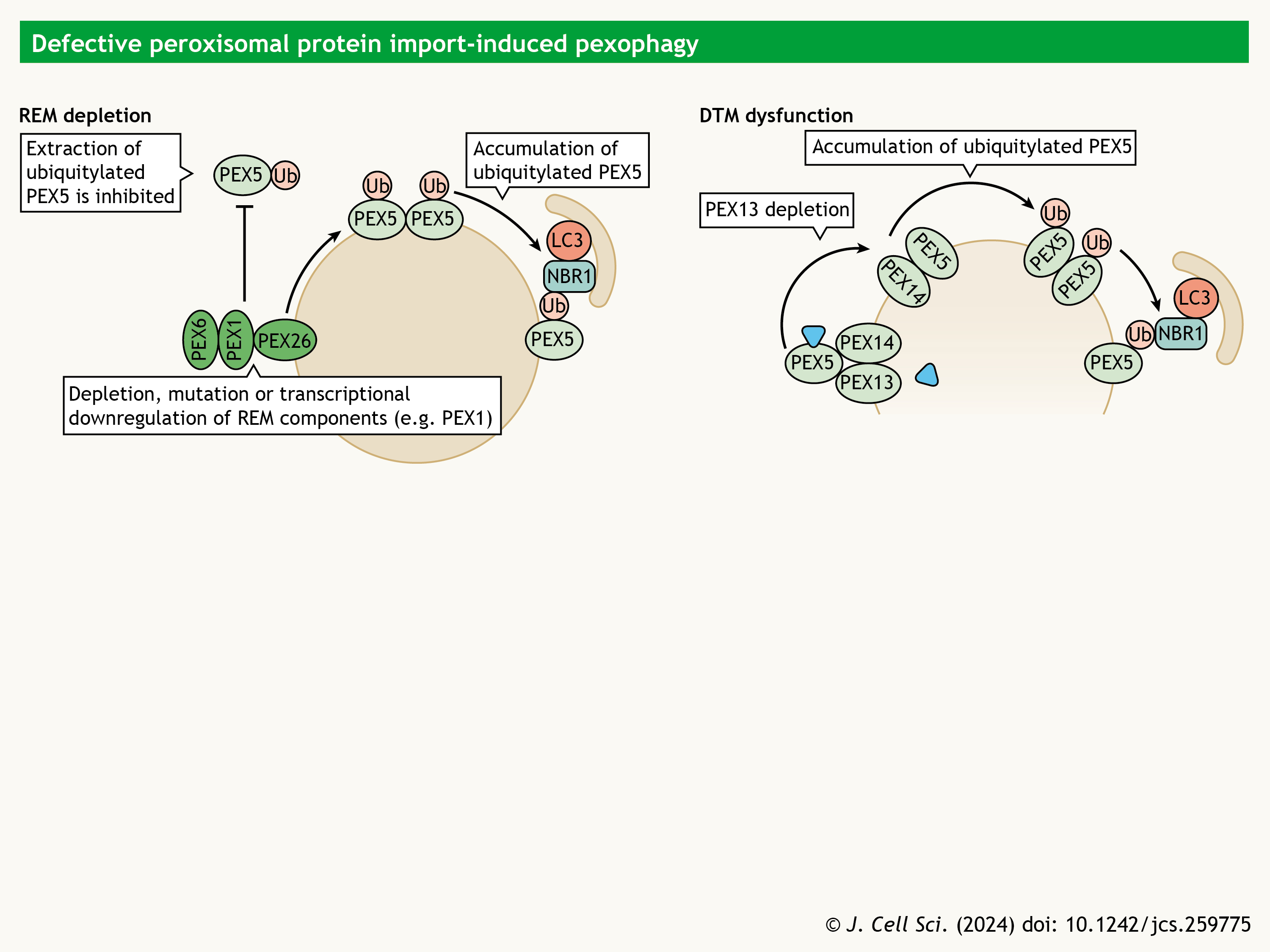

Supplement: Panel 6. Defective peroxisomal protein import-induced pexophagy [file joces-137-259775-s7.jpg]

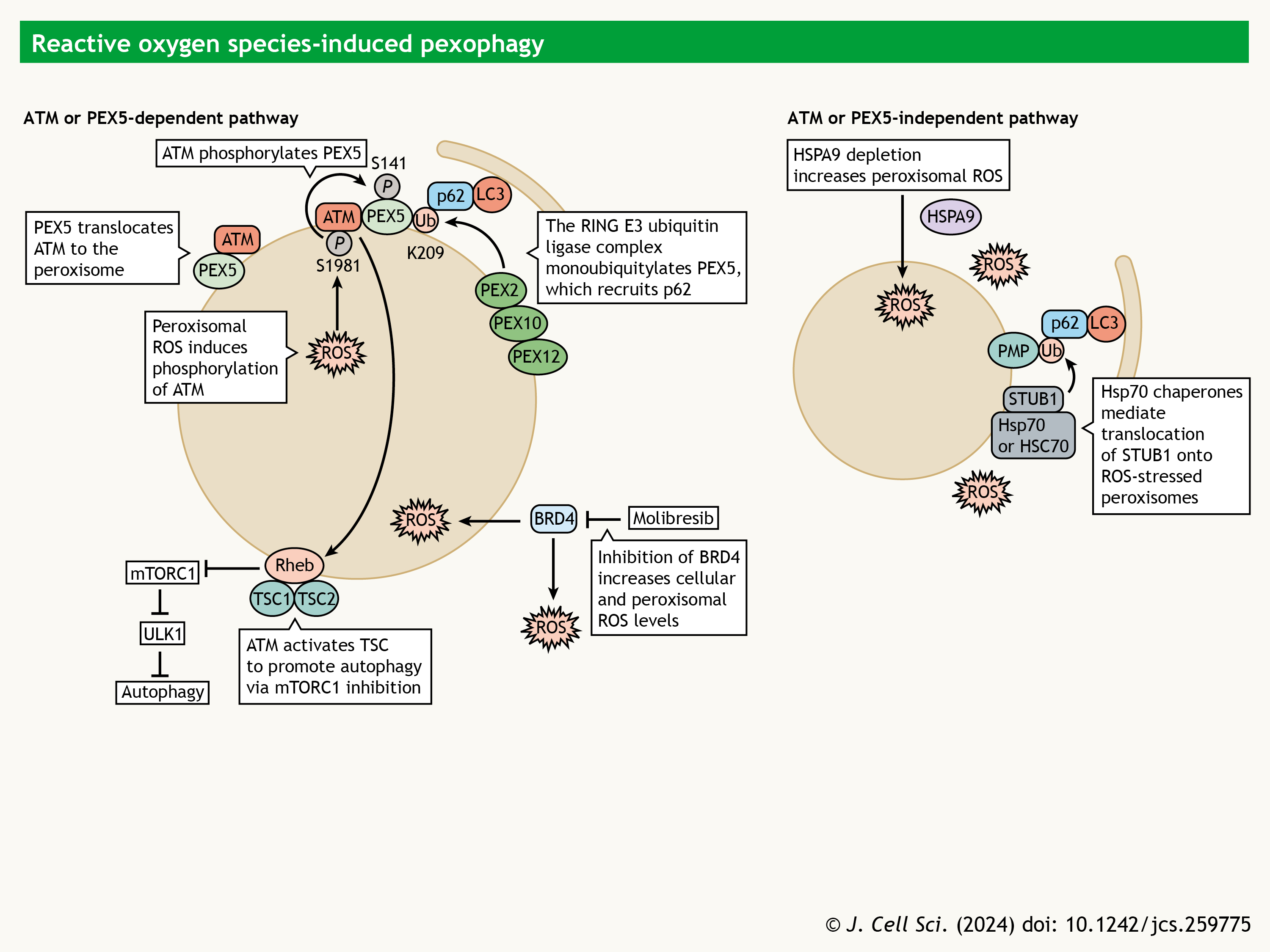

Supplement: Panel 7. Reactive oxygen species-induced pexophagy [file joces-137-259775-s8.jpg]

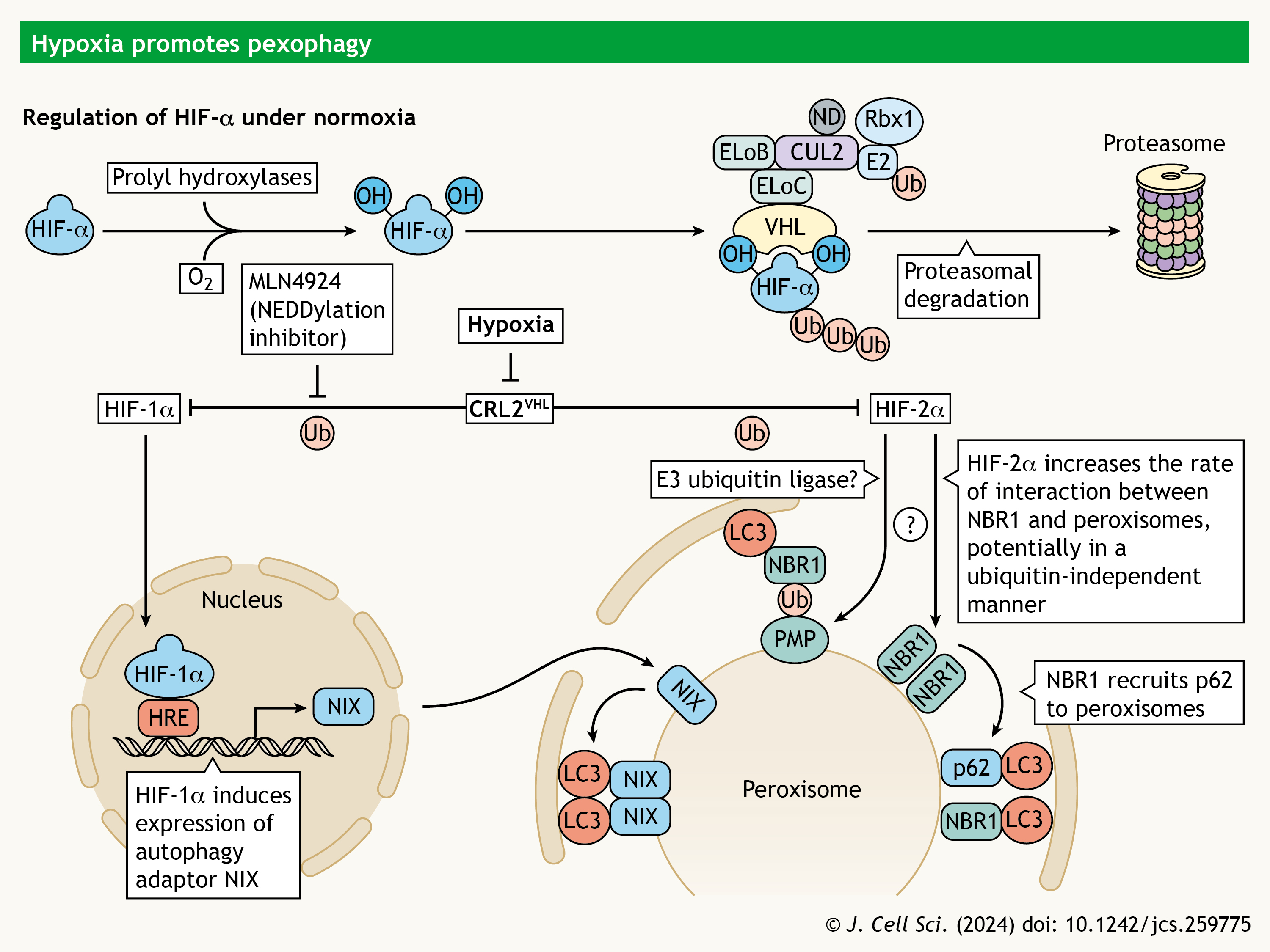

Supplement: Panel 8. Hypoxia promotes pexophagy [file joces-137-259775-s9.jpg]

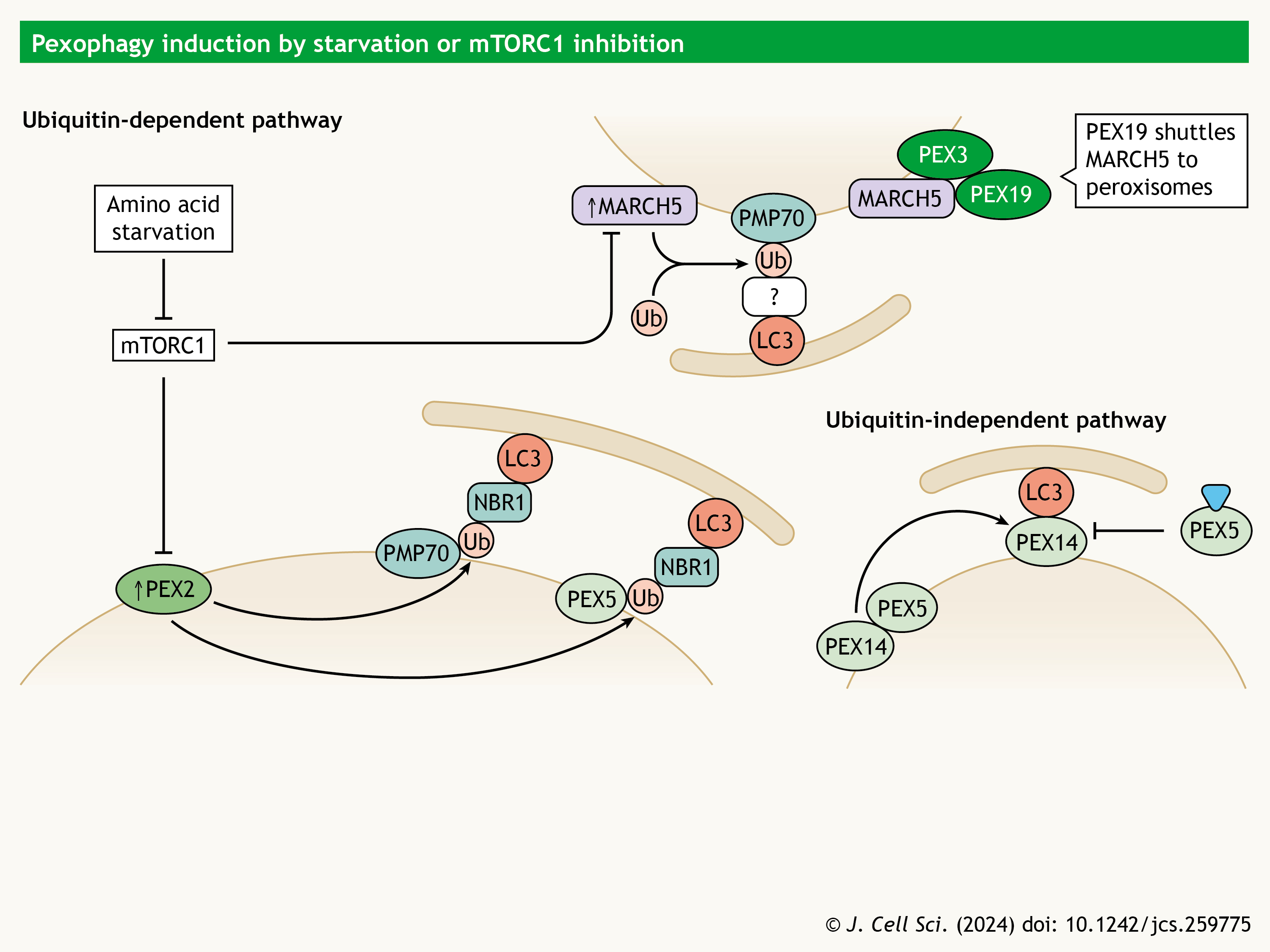

Supplement: Panel 9. Pexophagy induction by starvation or mTORC1 inhibition [file joces-137-259775-s10.jpg]

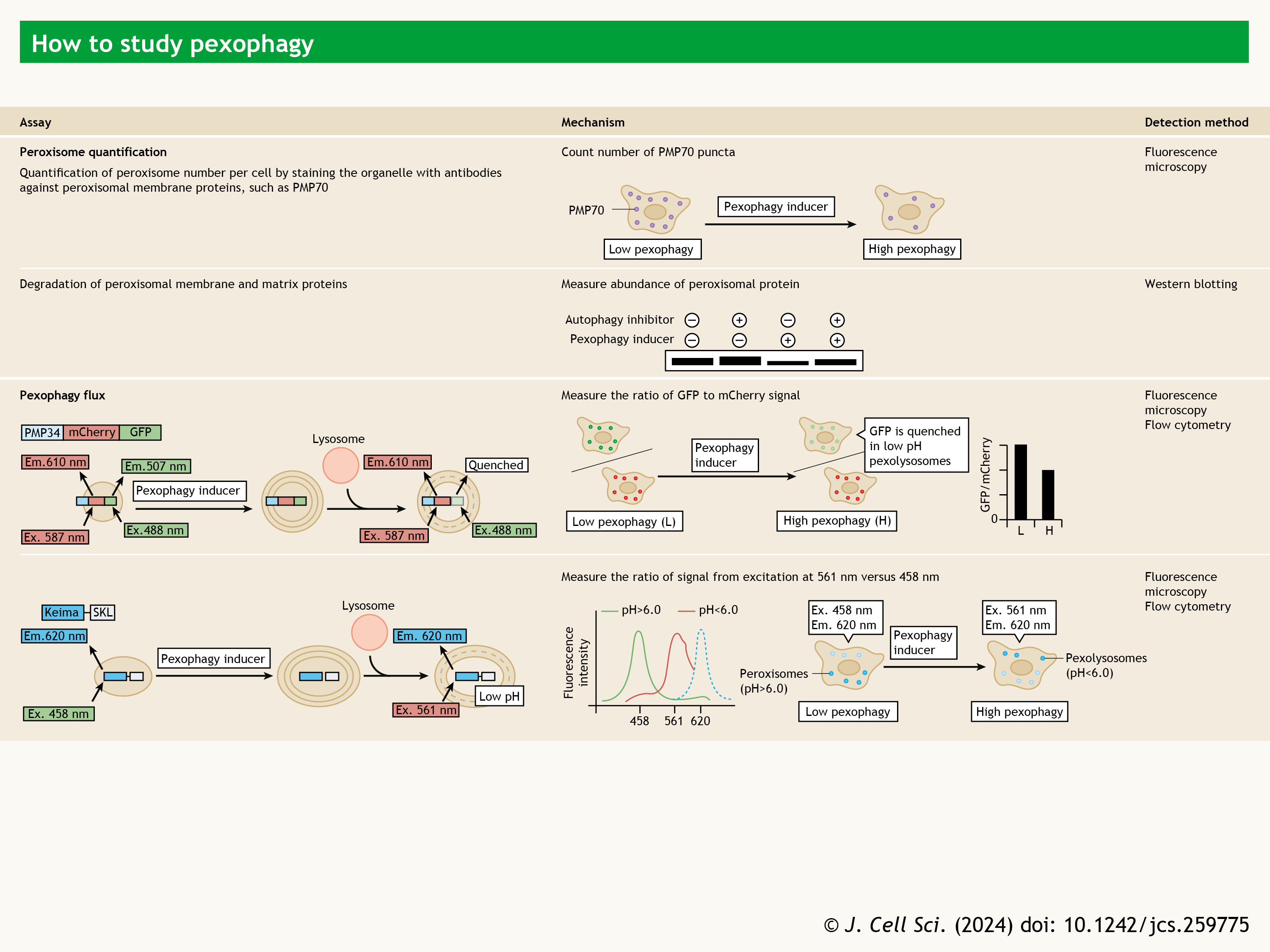

Supplement: Panel 10. How to study pexophagy [file joces-137-259775-s11.jpg]

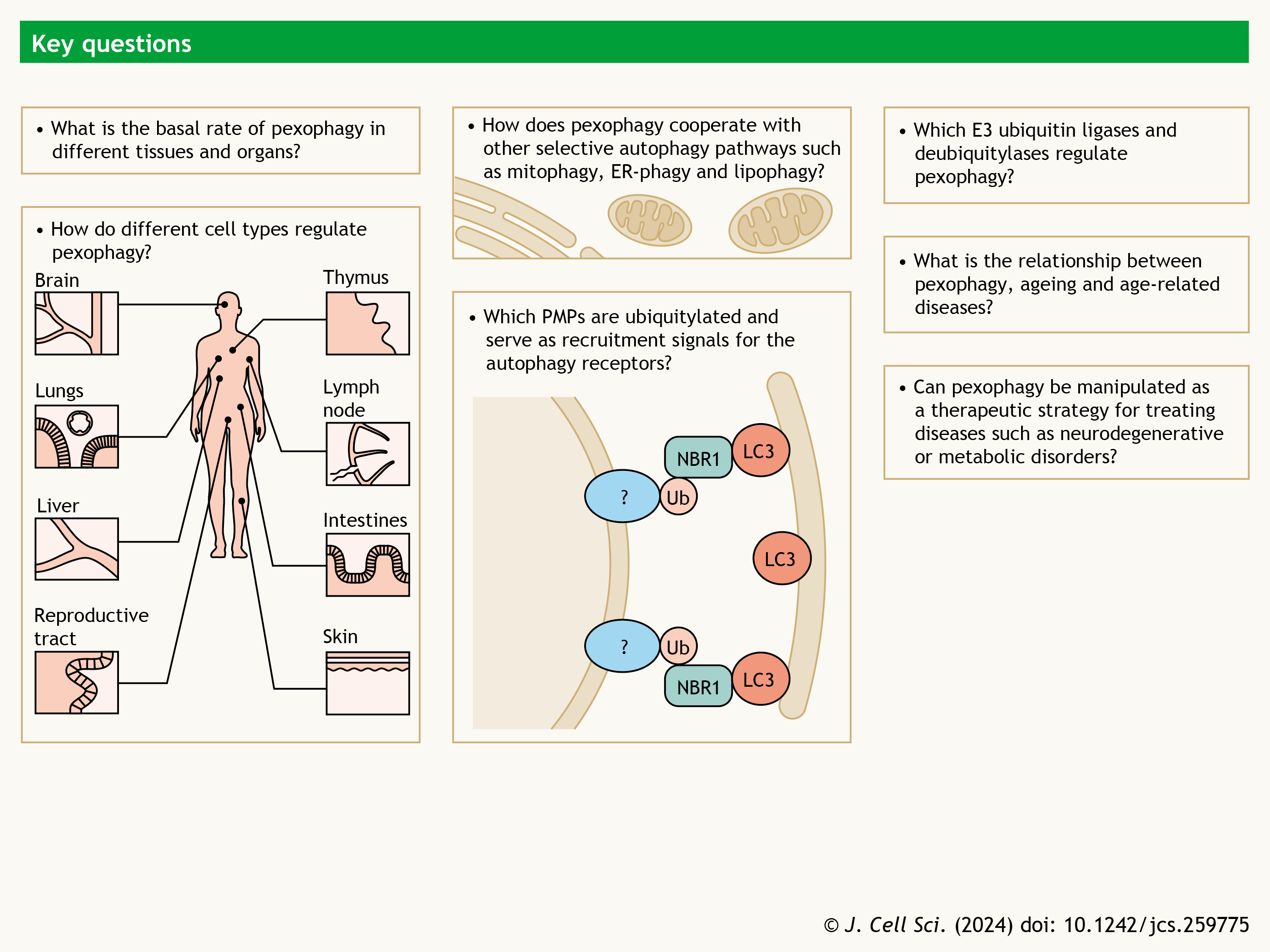

Supplement: Panel 11. Key questions [file joces-137-259775-s12.jpg]
